# MISSISSIPPI STATE UNIVERSITY LEVERAGES LAND-GRANT MISSION TO PURSUE AGE-FRIENDLY STATUS

**DOI:** 10.1093/geroni/igae098.1759

**Published:** 2024-12-31

**Authors:** David Buys, Christine Jackson, Ra’Sheda Boddie-Forbes, Jamie Dyer, Brent Fountain, Gary Jackson, Margaret Ralston, Susan Seal

**Affiliations:** Mississippi State University, Mississippi State, Mississippi, United States; Mississippi State University, Mississippi State, Mississippi, United States; Mississippi State University, Mississippi State, Mississippi, United States; Mississippi State University, Mississippi State, Mississippi, United States; Mississippi State university, Mississippi State, Mississippi, United States; Mississippi State University, Mississippi State, Mississippi, United States; Mississippi State University, Mississippi State, Mississippi, United States; Mississippi State University, Mississippi State, Mississippi, United States

## Abstract

**Introduction:**

Mississippi is experiencing outmigration and a decreasing birthrate, resulting in an aging population, which is of growing concern across sectors. The state’s Department of Health led an Age-Friendly Public Health System application and is facilitating a broader Age-Friendly Ecosystem Initiative. As a member of the ecosystem advisory board, MSU was asked to lead the state in pursuing Age-Friendly University (AFU) status.

**Methods:**

The University representative on the taskforce worked with MSU Divisions of Academic Affairs and Access, Opportunity, and Success (AOS) to assemble a working group to consider this opportunity. The group consisted of individuals from the Office of Outreach and Engagement, Interdisciplinary Studies, the Gerontology Education Program, the new College Of Continuing and Professional Studies and Cooperative Extension. The group 1) reviewed the AFU principles, 2) assessed where MSU has current initiatives that align with AFU goals, 3) considered where there are opportunities to develop new AFU-relevant work, 4) developed a proposal for the Provost and President, and 5) prepared and submitted an application to the AFU Global Network (AFUGN). Throughout the process the group has centered the unique role of the land-grant university mission in meeting the needs of the older adults. Results and

**discussion:**

Upon acceptance of the application by the AFUGN in August 2023, the Division of Access, Opportunity, and Success agreed to manage the designation as a convening body and “conscience” for the University. AOS convenes the working group monthly, promotes AFU-relevant activities, and monitors activities for AFUGN required reporting.

